# Fair Transmission of Individual Signals and Formation of Mainstream Information: Evidence from Herd Behaviours in Emergencies

**DOI:** 10.1155/2022/8229956

**Published:** 2022-08-24

**Authors:** Xintong Wu

**Affiliations:** School of Economics and Management, Beijing University of Chemical Technology, Beijing, China

## Abstract

Risk society is full of emergencies, accompanied by uncertainties and losses. Under emergencies, controlling herd behaviour is challenging due to more interactions and changes among individuals. This research establishes Bayes conditional probability models to explain the fair transmission of individual signals and individual decision-making after receiving others' signals. The simulation shows the following conclusions: first, each individual has a fair chance to influence the mainstream information; second, the order in which individuals make decisions during an emergency affects the difficulties and likelihood of making a rational decision; third, the high authority of information can become mainstream and guide individual behaviour; and fourth, two individual characteristics, including risk appetite and personal experience, are important in the fair transmission of individual signals and formation of mainstream information. According to the findings, this research proposes two strategies, including interfering with information and controlling existing key opinion leaders to control the mainstream information within a group in emergencies. These two strategies are proved to be useful in detecting and preventing approaches to alleviate individual herd behaviour, which should be monitored and controlled in machine learning models for individual behaviour simulation and prediction. Compared to previous research that focuses on media and public opinion in emergencies, this research focuses on a specific type of information (i.e., individual decision-making and actions) on the individual level and its effects on herd behaviours within the group. This research complements the explanation of the micro-mechanism of how individuals receive information and make decisions and actions.

## 1. Introduction

In recent years, there have been many large-scale herd behaviours following emergencies, which have been seen as secondary risks arising from the emergencies. The coming of risk society, proposed by Beck in 1992 [1], has become a consensus in safety engineering and management. In this society, the increasing emergencies that are accompanied by personal and property damage arise academic attention on technical controls, such as automatic control techniques, monitoring equipment, and technical improvement. Worse still, under such extremely high pressure [2], individuals may easily lose their rationalities and make irrational decisions. Also, a lot of individuals even give up their own choice and follow the group decisions, without any individual rational decisions. At this time, herd behaviour occurs [3]. Individual herd behaviour can further amplify the hazards of emergencies and even cause wider social impacts. For example, the rapid spread of public safety risks since the outbreak of COVID-19 has led to widespread social panic. This panic pressure forces consumers to overreact to the widespread information that the epidemic might lead to shortages of supplies, leading to a rush to buy related but unnecessary supplies (e.g., over-hoarded masks, disinfectants, household medicines, etc.).

Under the context of emergencies, there is a specific decision-making condition—mainstream information—that could trigger individual herd behaviours. Individuals' herd behaviour can make a great influence on emergency control [4], which directly relates to whether the rescue actions and plans can be implemented effectively. Unlike physical technologies and equipment, herd behaviour is affected by information dissemination and individual psychological bias [5]. Among the diversity of information, mainstream information has a huge impact on individual behaviour. Mainstream information refers to the option that has the largest proportion of decision options made by a specific number of people in a specific time period [6, 7]. On the one hand, information dissemination always carries implications about proportion, which is mainstream information in essence. On the other hand, individual psychological bias often originated from mainstream information in a specific group, which is usually proved to be lopsided and overwhelming. When individuals make decisions in emergencies, they are embedded in a decision context in which individuals are heavily exposed to the decisions and actions of others [8]. It is confirmed that a large amount of external information might exceed personal information processing capabilities, and their own decisions are influenced by others [9]. Previous literature has proved that mainstream information significantly is aggregated and could influence how individuals evaluate alternative options and perceive risks [10, 11]. An example is that in July 2021, a fire broke out in a food processing plant near the capital of Bangladesh. After the fire, many people accepted the seemingly trustworthy plan of jumping from the roof to escape as suggested by several experienced workers, resulting in death. This suggests that mainstream information plays a critical role in herd behaviour in emergencies and requires empirical research to further investigate its underlying mechanisms.

Information, especially mainstream information, is essential for rational decision-making; however, two issues have not been taken seriously. Firstly, existing decision modelling and machine learning on irrational behaviour have not taken into account the corresponding decision processes [12]. While prior research on decision models has simulated the irrational behaviour of individuals, they take the actual outcome of the irrational behaviour as a starting point for modelling and attempt to propose methods to detect, prevent, and/or alleviate undesired bias [12, 13]. This approach is unable to describe and model the irrational decision-making process of individuals. In fact, individuals' decision-making under emergencies is progressive: each individual constantly weighs the decisions already made by others, compares them with his or her existing knowledge, and endeavours to make decisions that he or she believes to be optimal. Therefore, it is necessary to demonstrate individual sequential decision-making processes in modelling. For example, the Bayesian conditional probability model is a suitable process model. Second, there is a feature that a fair transmission of individual signals exists in emergencies. Under emergencies, due to the urgency of making quick rescue and escape decisions in such a short period, individuals will view all previous decisions equally. In other words, everyone has an equal ability to influence public opinion in the fair transmission and individual characteristics deserve to be discussed. It means that the impact of endogenous uncertainties is significant and individuals might change their decisions after receiving mainstream information and transmit their own opinions to others. Individuals with different types of risk appetites may not accept existing mainstream information and instead become key opinion leaders and sources of mainstream information [11].

To fill these two gaps, this research focuses on the literature on the process of mainstream information and explores the following questions: how mainstream information arises, whether mainstream information can dominate in a specific group, and what the process of herd behaviour is caused by mainstream information. Based on the literature about herd behaviour and the process of mainstream information in emergencies, this research builds two models, including a Bayes conditional probability model about how individual signals lead to mainstream information, and a probability model to describe the decision-making process of that whether the individual signal can be accepted by a specific group, which contains members with different types of risk appetite and leads to rational/irrational decision-making. Moreover, the series of numerical simulations about the process of herd behaviour and rational decision-making provide evidence to support our arguments and help to expand the understanding of the role of mainstream information in emergency control and its corresponding control techniques in emergencies.

This research makes three contributions. First, emergencies provide contextual conditions for the fair transmission of individual signals. In emergencies, people need to make decisions in a period of time [14]. Consequently, they gather as much information as possible and magnify the likelihood of some small probabilities in order to make relatively rational decisions. In this case, some information with a low or medium level of authority will not be able to guide individual behaviour [12, 13]. Second, different from other studies on public opinion control in emergencies, this research focuses on a specific type of information in emergencies, that is, the information on individual decision-making and the corresponding actions. Peoples always make decisions depending on the decisions or actions of others in some emergency scenarios and even neglect the information they already have [11]. Therefore, the information on individual decision-making and actions that are available in a group is an important source of information in emergencies. Third, information on individual decision-making and actions is on the individual level, and its arising, acceptance, and transmission process of mainstream information could complement the micro-mechanism of how individuals receive information and make decisions and actions [5].

## 2. Related Work

### 2.1. Herd Behaviour in Emergencies

Herd behaviour is derived from animals moving and foraging in groups. Social scientists introduce it into research and use it to describe the phenomenon that individuals in groups tend to align with the majority of the group members on consideration, thinking, perception, and action [14]. This specific alignment will be widely spread and be accepted within the group. In society, common herd behaviours include seasonal fashion, investment boom, the festival rush, escaping in emergencies, rumours, *etc*. These are the results of the individual's irrational/rational behaviour being transmitted in society. Dirk Helbing, Illés Farkas, and Tamás Vicsek published a paper titled *Simulating dynamical features of escape panic* on *Nature* in 2000, which simulates the escape panic process and its characteristics. They find that in the escape process of emergencies, such as fire, collapse, and gas diffusion, panic and stampede happen easily. Therefore, they propose the term “hybrid herd instinct” of individuals and groups [15] to explain why escape panic occurs. The model indicates that both individual behaviour and herd behaviour can contribute to positive results. As for individual behaviour, an individual's successful escape is contingent and can help to find an escape exit. Once an individual finds a correct escape direction and it is not completely closed, the direction would be imitated immediately by others [16]. As for herd behaviour, it identifies and decides a shared escape direction for the group. Meanwhile, the three researchers summarise the potential results of herd behaviour in emergencies, including higher convergence rate, more pushing and rubbing among individuals, overlap congestion, and more neglected and abandoned escape directions.

The herd behaviour caused by mainstream information in emergencies describes the behaviour bias of individuals and groups, and its influence on emergencies. According to the literature on emotional contagion, herd behaviour is a kind of emotional rush in a group caused by individual emotion, and it is more easy to be driven in a spontaneous, disorganised, and uncertain situation [17]. From previous research, there are several reasons to explain why this behaviour bias is much more likely to occur. First, behaviour bias is caused by insufficient procedures and routines. The daily procedures and routines are usually unsuitable for emergencies that are nonprocedural and paroxysmal [18]. Under normal context, behaviour decisions and actions highly depend on previous experience and actual situations, which could make apriority cognition on actual situations and affect judgments. However, in the case of unexpected events or contingencies, individuals cannot draw on previous experience to process information or issues. This makes it difficult for individuals to come up with optimal solutions when faced with such contingencies for the first time [19, 20]. Secondly, behaviour bias is caused by the framing effect [21, 22]. The framing effect describes how individuals are influenced by situations and expressions. For example, the response of security inspectors to exceptional conditions is affected by the presentation of forecasts and alarm information, and even makes different action decisions. That is, external situational pressures would interfere with individuals' decision-making and thus affect their ability to make optimal decisions [23, 24].

### 2.2. Fair Transmission of Individual Signals and Mainstream Information in Emergencies

Previous research has suggested that once mainstream information is formed, it can be overwhelming and have a significant impact on an individual's decision-making. Usually, information from the official government and media has been unconditionally identified as mainstream information. This assumption is assumed to be inapplicable in emergency scenarios. As individuals need to make quick escape/rescue decisions in a short period of time, they will view each previous decision equally. In essence, mainstream information is a kind of information that most people believe to be correct and takes up a large proportion of information content [25]. Gradually, mainstream information would change during the fair transmission process, and due to that many alternative information would also be accepted by individuals. If the proportion of alternative information exceeds others, it will transfer from alternative to mainstream [14].

Under fair transmission of individual signals, individual characteristics should be emphasised. In emergencies, there are some psychological factors affecting mainstream information. The first is the availability effect. It refers that individuals tend to make decisions depending on the integrality of available information determined by media coverage and knowledge dissemination about emergencies [14]. Once the negative information is lacking or insufficient, individuals tend to consider that emergencies are less likely to occur. The second is the primacy effect. Compared with subsequent information, prior experience or priority input information has a greater impact on decision-making. In safety management practice, equipment usually needs to be constantly monitored and updated, which could constantly generate real-time data and surrounding environment information. However, operators and monitors often make judgments from the data which are collected for the first time and ignore the current specific situation [26]. The third is the recency effect. Recent information plays an important role in short-term memory, thus influencing forward judgment and often ignoring prior experiences. In practice, once no production safety accident has occurred recently, the operators may neglect the risks, have no safety production behaviour and control awareness, and be mentally negligent [23].

There are two types of herd behaviour. One is information-dependent herd behaviour. The term, “informational cascades,” is proposed to explain the phenomenon in buying decisions and seasonal fashion [26, 27]. They put forward that information flow leads decision-makers to ignore the previous information and follow peer behaviour. However, information flows are vulnerable, because those individuals in a group will quickly converge to a consistent action based on information flows, yet it can lead to a massive behaviour shift due to a tiny new information shock. This phenomenon is described as informational cascades. Then, they establish the model, including taking into account their own information, received information, observation, and the reference signal. Another one is reputation-dependent herd behaviour [14, 26]. Extant research deliberates on rational herd behaviour in the financial market and finds that agents' reputations can make direct profits to banking and transaction liquidity [28]. Some empirical research proves that some parts of herd behaviour are inefficient, for that investors often imitate other decisions with high reputations. Once investors find that the return of the investment project is uncertain, they will tend to make a consistent judgment with the group for buck-passing. Once the return on the investment project is found to be stable, it is inclined to make a reverse decision to improve its reputation.

## 3. Preliminaries

The preliminaries describe the generation process of mainstream information, before herd behaviour. It is divided into two sub-processes, that is, the arising of mainstream information and the acceptance of mainstream information.

### 3.1. The Arising of Mainstream Information

According to the effect of informational cascades, it is confirmed that under specific circumstances and situations, individuals tend to rely too much on others' information or behaviours as a reference and even ignore the information they already have [29, 30]. In emergencies, operators will make wrong decisions due to insufficient safety rescue knowledge, too much confusion, wrong understanding, and insufficient alarm content. Moreover, due to the crisis pressure, lack of training, and emergency drills, operators are afraid to make quick judgments, even ignore the on-site situation, and rely too much on mainstream information. This research establishes a model exploring how mainstream information arises which is a precondition to simulating the herd behaviour process.

Scenario: a serious emergency occurs, and there are two rescue plans, A and B. Individuals have to make an either-or choice in turn.
The decision order: individual decision order is exogenous and does not affect the emergency.
The decision of rescue plans: There are two rescue plans, A and B, and there is no clear distinction between the two plans. When an individual is completely rational, the probability of choosing each plan is 50%.
The source of information flow: each operator has information stock, from previous work experience, safety training, safety education, emergency drills, *etc*. After the occurrence of emergencies, individual information stock would be affected by public information, messages from other workers, and alarm information. All these are components of information flow.
The process of decision-making: individual decision-making is based on acquired information stock, information flow, and evaluation of on-site conditions. Once an individual chooses the A or B plan, it makes the corresponding signal, that is, ***I***_***A***_ or ***I***_***B***_, to other surroundings.
Modelling algorithm: this research adopts the Bayesian conditional probability algorithm, a widely used classification algorithm in machine learning, to describe the individual decision process. Bayesian decision theory is the basic method for implementing decisions in a probabilistic framework. His algorithm has been widely used for classification tasks and is based on the principle that in an ideal situation where all relevant probabilities are known, and Bayesian decision theory considers how to select the optimal class label based on these probabilities and misclassification losses. This classification principle is similar to the individual decision-making process due to the task of individual decision-making to find a decision that minimises the sum of the conditional risks of all options. This study applies Bayesian algorithms to individual decision-making in emergencies as a way of describing the decision-making process.
The process of information dissemination: there are other persons around who observe the previous decision-making and receive the signal, which means that a person who makes decisions later in the decision-making order would receive more signals that disrupt their rational decision-making. If they observe that the last person chooses A, then the probability of receiving ***I***_***A***_ is ***r***, and receiving ***I***_***B***_ is (1-***r***). Similarly, if he observes that the person chooses B, then the probability of receiving ***I***_***B***_ is ***r***, and receiving ***I***_***A***_ is (1-***r***).
  (1) $$\begin{array}{c} P\left(I_{A}|A\right)=P\left(I_{B}|B\right)=r\in \left(0.5,1\right), \end{array}$$
  (2) $$\begin{array}{c} P\left(I_{B}|A\right)=P\left(I_{A}|B\right)=1-r. \end{array}$$
Emergencies treatment: individuals need to obey the superior's rescue plan, and after a rescue plan takes the mainstream, the superior will adopt the plan.

The first decision-maker (*M*_1_) makes decisions entirely according to his own information stock, and the probability of choosing A or B is 0.5, being that ***P***_***1***_***(A)*** = ***P***_***1***_***(B)*** = 0.5.

The second decision-maker (*M*_2_) makes decisions according to his own information stock and M1's choice. If *M*_2_ receives the signal ***I***_***A***_, the probability of choosing A is

(3) $$\begin{array}{c} P_{2}\left(A|I_{A}\right)=\frac{P\left(I_{A}|A\right)\times P\left(A\right)}{P\left(I_{A}|A\right)\times P\left(A\right)+P\left(I_{A}|B\right)\times P\left(B\right)}=\frac{r\times 0.5}{0.5r+0.5\left(1-r\right)}=r\in \left(0.5,1\right). \end{array}$$

If *M*_2_ receives the signal ***I***_***B***_, the probability of choosing A is

(4) $$\begin{array}{c} P_{2}\left(A|I_{B}\right)=\frac{P\left(I_{B}|A\right)\times P\left(A\right)}{P\left(I_{B}|A\right)\times P\left(A\right)+P\left(I_{B}|B\right)\times P\left(B\right)}=\frac{\left(1-r\right)\times 0.5}{0.5\left(1-r\right)+0.5r}=1-r\in \left(0,0.5\right). \end{array}$$

The third person (*M*_3_) can not only receive the signals from *M*_1_ and *M*_2_, but also observe the consistency between the signal from *M*_1_ and the choice of *M*_2_. At this point, *M*_1_'s signal becomes M3's information stock. He makes decisions based on his own information stock and signals sent by M_2_. The decision-making process is as follows: (a) recalling information stock and (b) observing whether the previous decision-maker's behaviour is consistent with information stock. For example, when *M*_3_'s information stock contains ***I***_***A***_ and he observes that *M*_2_'s choice is A, then *M*_3_ would believe that A is the optimal choice. When *M*_3_'s information stock contains ***I***_***B***_ and he observes *M*_2_'s choice is A, it is equivalent to receiving two independent information, and the corresponding probability of choosing A or B is random, **p**.

The fourth person (*M*_4_) makes decisions based on information stock and *M*_3_'s signal. The decision-making process is as follows: (a) recalling information stock and (b) observing the last person's action and recognising the mainstream information.

Before *M*_4_ makes decisions, the probability of generating ***I***_***A***_ mainstream information is

(5) $$\begin{array}{c} P\left(I_{A}\right)=P_{1}\left(A\right)\times P_{2}\left(A|I_{A}\right)\times P_{3}\left(A|I_{A}\right)+P_{1}\left(B\right)\times P_{2}\left(A|I_{B}\right)\times p+P_{1}\left(A\right)\times P_{2}\left(A|I_{A}\right)\times P_{3}\left(B|I_{A}\right)+P_{1}\left(A\right)\times P\left(B|I_{A}\right)\times p=r^{2}+\frac{1}{2}-\frac{1}{2}r-\left(1-r\right)p>0. \end{array}$$

And the probability of generating ***I***_***B***_ mainstream information is

(6) $$\begin{array}{c} P\left(I_{B}\right)=P_{1}\left(B\right)\times P_{2}\left(B|I_{B}\right)\times P_{3}\left(B|I_{B}\right)+P_{1}\left(B\right)\times P_{2}\left(A|I_{B}\right)\times p+P_{1}\left(A\right)\times P_{2}\left(B|I_{A}\right)\times p+P_{1}\left(B\right)\times P\left(B|I_{B}\right)\times P_{3}\left(A|I_{B}\right)=r^{2}+\frac{1}{2}-\frac{1}{2}p-\left(1-r\right)p>0. \end{array}$$

*M *_4_'s probability of choosing plan A is

(7) $$\begin{array}{c} P_{4}\left(A|I_{A}\right)=P\left(I_{A}\right)\times P\left(A|I_{A}\right)+P\left(I_{B}\right)\times P\left(A|I_{B}\right)=2r^{2}+1-r-2\left(1-r\right)p>0. \end{array}$$

*M *_4_'s probability of choosing plan B is

(8) $$\begin{array}{c} P_{4}\left(B|I_{A}\right)=P\left(I_{A}\right)\times P\left(B|I_{A}\right)+P\left(I_{B}\right)\times P\left(B|I_{B}\right)=2r^{2}+1-r-2\left(1-r\right)p>0. \end{array}$$

Since *M*_5_'s decision-making, the mainstream information arises and individuals will follow it and be without conditional probability. The corresponding probability of generating mainstream information is

(9) $$\begin{array}{c} PI=P\left(I_{A}\right)\times P_{4}\left(A|I_{A}\right)+P\left(I_{B}\right)\times P_{4}\left(A|I_{B}\right)=2r^{2}+1-r-2\left(1-r\right)p. \end{array}$$

### 3.2. The Acceptance of Mainstream Information

In the above model, the main external factor of herd behaviour is information. Once someone selectively accepts this information, mainstream information arises. The mainstream information reflects that certain information occupies a large proportion, which essentially indicates that the group stress has been formed, which can affect individual intuition based on the framing effect. In actual emergencies, there are other kinds of information, such as emergency reports, alarm information, knowledge stock, and external signals. This excessive and complex information makes people tend to transfer self-decision-making power to others and comply with others' behaviour as their own standard. People usually choose the behaviour with a higher proportion as their own behaviour. Each plan, however, has costs and benefits. Rationally, whether mainstream information can be accepted depends on the cost and benefits of plans.

The probability of choosing plan A is **p**, correspondingly, the probability of choosing plan B is $\bar{p}$, and the choice is either-or. The proportion of the population who choose A is *λ*, and **p** is a function of *λ* [16]:

(10) $$\begin{array}{c} P_{A}=p=f\left(\lambda \right). \end{array}$$

The baseline of value judgment is prospect theory [17]. ***x*** represents the possible loss of life and property of plan A, and ***y*** represents the potential benefits of other plans (also call it opportunity cost). Thus, the expected value of plan A is

(11) $$\begin{array}{c} VA=\pi \left(\lambda \right)V\left(x\right)+\pi \left(1-\lambda \right)V\left(y\right). \end{array}$$

Thereinto,

(12) $$\begin{array}{c} V\left(0\right)=0,\pi \left(0\right)=0,\pi \left(1\right)=1. \end{array}$$

(13) $$\begin{array}{c} V\left(x\right)<0,V\left(y\right)>0. \end{array}$$

Whether an individual accepts others' signals and mainstream information is influenced by the types of risk attitudes, including risk neutral, risk aversion, and risk appetite. The types of risk attitudes will change during emergencies. In this model, different types of risk appetite will lead to different actions and difficulties of mainstream information being accepted, which is shown in Table 1. The behaviour of risk appetite could alleviate the blindness of herd behaviour to some extent, but hesitant individual decision-making processes can affect the timing of plans. Risk aversion can lead to herd behaviour; however, the consistency and rapid convergence of herd behaviour can ensure the effective implementation of plans. Therefore, for different safety accident scenarios, individuals of different risk appetite types should be concerned.

## 4. Simulation of the Herd Behaviour Process and Decision-Making

After showing the forming process and acceptance process of mainstream information, this research designs a simulation model to show the herd behaviour in emergencies.

### 4.1. Running Mechanism and Process

  Setup: there are 150 people (agents) in emergencies, where 10 people hold escape plan A and the proportion of A is 1/15 (*λ*_*t*=0_  =  1/15).
  State mechanism: people move around randomly and are in one of three states, (1) have not made decisions, but are susceptible to mainstream information (labelled “involve” in green), (2) have chosen escape plan A (labelled “accept” in red), and (3) have not made decisions, but stick to their own points (labelled “hesitate” in grey).
  Mechanism of population change: with the evolution of emergencies, there will be new victims involved in emergencies. Once an individual dies or succeeds to escape, he disappears in emergencies, and the population declines. When the population declines and falls significantly, this may indicate either the severity of the emergencies (a significant increase in deaths) or the fact that the emergencies begin to dissipate. The number of involved populations (green agents) represents the complexity and chaotic degree of herd behaviour. Moreover, in order to ensure that emergencies do not expand excessively (that large emergencies lead to an escalation of emergency response, making the model conditions inapplicable), the upper limit of the population is set to 300. Once the number of people exceeds 300, no new victims will be involved. This setting influences the value change of *λ*.
  The density of the population: the initial density of the population is 150, and it affects acceptance and contact frequency. This setting also influences the value change of *λ*.
  Time window: the duration of one single cycle is 200 times. If a significant pattern of population change is detected, the simulation model would stop at the end of the cycle; otherwise, it would run for the next cycle.
  Acceptance (*p*): it shows the authority of escape plan A or the intensity of the signal ***I***_***A***_. Disseminate information will vary according to the mode of transmission. Common modes of transmission include behaviours, face-to-face conversations, media reports, etc. The authoritative and credible way will be more acceptable.

Then, the arising and acceptance process of mainstream information was coded into simulation models and set further to make the following parameter setting.

### 4.2. Parameter Setting

The main variables in the model are acceptance and chance to recover, which could be controlled in simulation tests. ***Acceptance (p)*** refers to the probability of an involved person's acceptance of escape plan A when a recipient of escape plan A comes into contact with the involved person (i.e., a red person being in contact with a green person). When the acceptance slider is set to 50, one of every two transmissions will be successful. The change (*θ*) shows the possibility of a change in risk appetite (such as risk aversion ⟶ risk appetite and risk appetite ⟶ risk aversion). It is possible that after they receive escape plan A, they will accept a new plan and their attitude will change. In all simulation tests, a balance is achieved among all parameters and there are two assumptions in these simulation models. First, the number of involved population (green agents) is sufficient in order to ensure enough potential receivers, which also demonstrates the spreading of emergencies. Second, the recipients of escape plan A (i.e., red agents) have access to the green agents. The design of the simulation is that two main variables (e.g., acceptance and change) are, respectively, controlled at three levels, and a total of 9 simulation tests are conducted (see Table 2).

### 4.3. Results

#### 4.3.1. The Results of the Information with Low Acceptance

These models describe the behaviours of the information with low ***acceptance***. In the first three simulation models, the value of ***change*** is set from 0% to 50%, to 80%; the value of ***acceptance*** is 20%. In these models, escape plan A is not the mainstream information in the population because the value of ***acceptance*** is only 20% and the proportion of recipients holding escape plan A is low. The corresponding parameter setting and characteristics of the simulation are summarised in Table 3.

**Figure 1:**
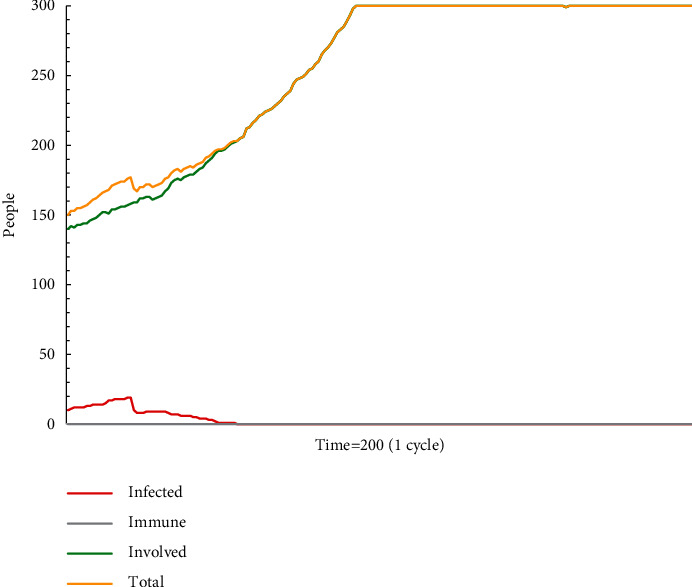
The status monitor of A1.

In simulation test A1 (see Figure 1), escape plan A makes a significant effect on group behaviours in a specific period, but it has not dominated in this group. As the populations die or are rescued, the proportion of recipients holding escape plan A gradually decreases. After escape plan A has spread for a period of time, it will naturally disappear in the diffusion in the group. When the model stops running, all people are involved but do not adopt escape plan A. According to the data, the attenuation of escape plan A occurs in No. 20 time, and the number of recipients is 19. For employees who lack experience and knowledge, such as new employees, they neither stick to their opinions nor readily choose to accept escape plan A, which has not yet dominated as the mainstream information, but are more inclined to hesitate without any action. As a result, in such situations, there are often delays in action and rescue plans that are not accepted and implemented in a timely manner.

**Figure 2:**
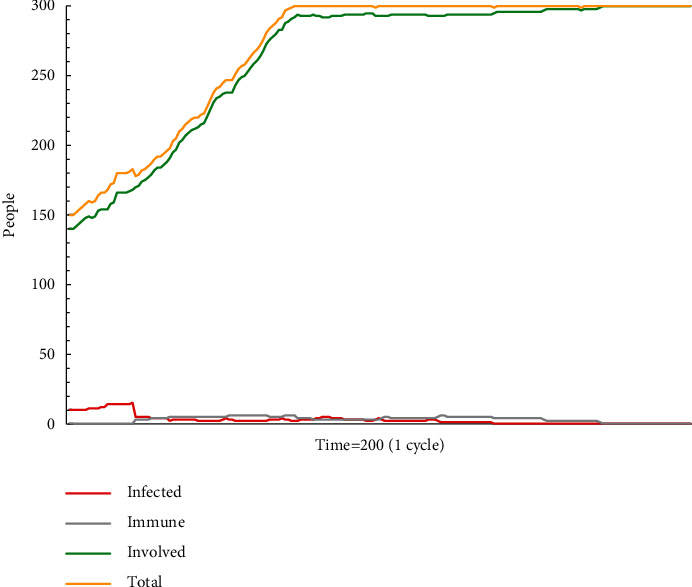
The status monitor of A2.

In simulation test A2 (see Figure 2), the escape plan A makes effects in a very limited time. The total number of agents and the green agents start to remain consistent at No. 74 time. All new victims adopt a wait-and-see strategy. The attenuation of escape plan A occurs in No. 20 time with the number of recipients of escape plan A being 15, and it drops fast. At the 23rd time, the number of recipients who accept escape plan A has been close to zero. The main reason why escape plan A spreads quickly and is not accepted is that it is not mainstream information. Also, as for employees who have some experience and knowledge, some of them will insist on their own opinions, which will interfere with the information conditions in this group. Under this condition, information that does not have authority is often difficult to gain mass acceptance through the diffusion process. Mainstream information is difficult to form quickly, and much information (even correct information) dissipates. Large-scale populations are hesitant and delay actions.

**Figure 3:**
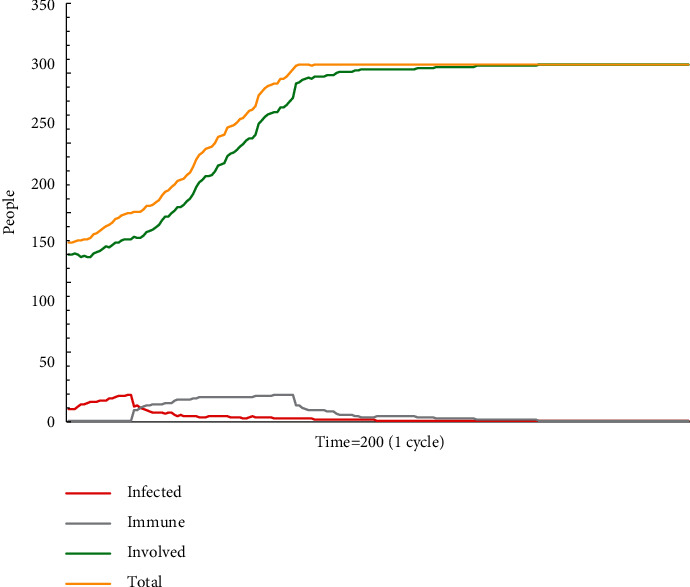
The status monitor of A3.

In simulation test A3 (see Figure 3), escape plan A makes an effect for a very limited period, but the period is extended. In this simulation model, individuals have abilities to judge the correctness of the escape plan and to make decisions. Even if they hold the idea that escape plan A is untrust, they are able to send a signal to the outside about their actions. This simulation model is applicable when there are a large number of experienced employees among the involved populations. They are able to correctly determine the truthfulness of various information, thus sending outward signals to select or exclude certain information. For some nonmainstream information, experienced employees can help identify false nonmainstream information and block further dissemination, as well as filter out the correct nonmainstream information to help their dissemination.

#### 4.3.2. The Results of the Information with Medium Acceptance

These models describe the behaviours of the information with medium ***acceptance***. In the middle three simulation models, the value of ***change*** is set from 0% to 50%, to 80%; the value of ***acceptance*** is 50%. In these models, since the value of ***acceptance*** is 50%, the probability of accepting escape plan A becoming the mainstream information is random. Even though escape plan A has been constantly diffused, the information contents are still complex and chaotic, and unable to form unified and convergent group behaviour, which leads that is difficult to coordinate actions. The corresponding parameter setting and characteristics of the simulation are summarised in Table 4.

**Figure 4:**
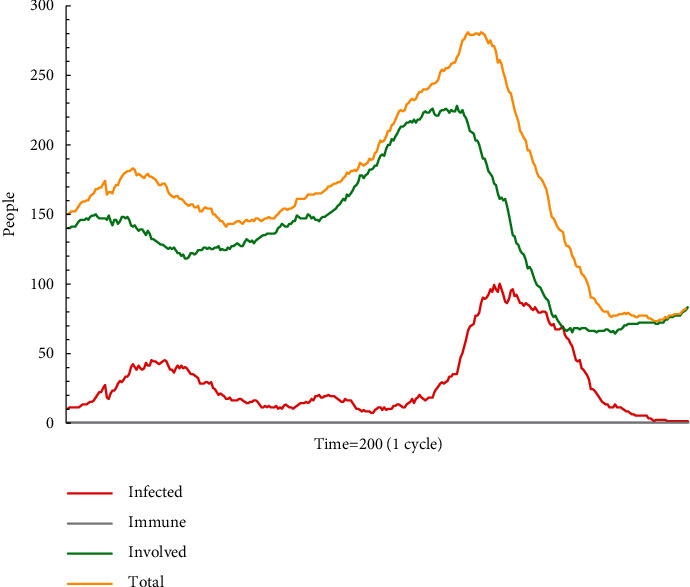
The status monitor of B1.

In simulation test B1 (see Figure 4), the number of agents in different states constantly changes, but the rank of proportions is fixed. The fluctuation of the proportion of recipients supporting escape plan A is based on the new agents whether to accept the mainstream information. As shown in Figure 4, after escape plan A has been diffused for some time, there is significant randomness in the proportion of recipients. It is difficult for new employees, who lack knowledge and experience, to judge the authenticity of such information. In other words, confusing information is uncontrollably spread among inexperienced individuals.

**Figure 5:**
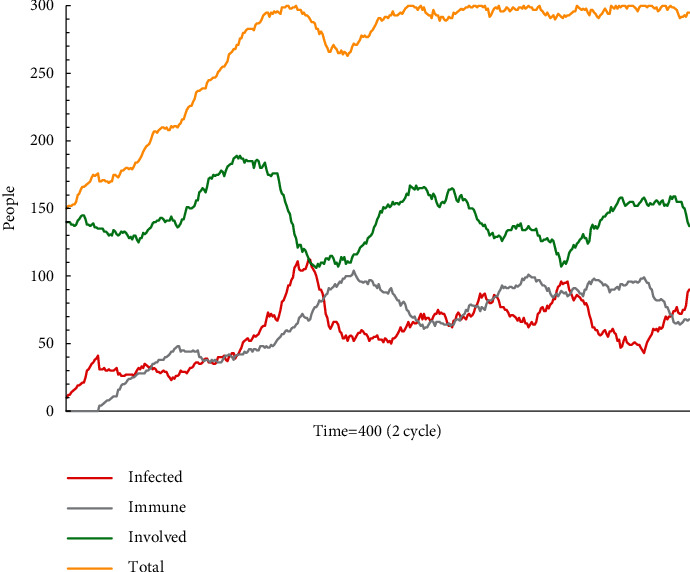
The status monitor of B2.

Figure 5 presents the results of simulation test B2. Since the probability of accepting escape plan A is 50%, it is not the dominant message in the group. Both the numbers of people who insist on their own opinions and who accept escape plan A change significantly, and the information situation in the group is confusing. This simulation model runs for two cycles before showing a fluctuation pattern: those who remain on the wait-and-see strategy dominate, while those who support escape plan A and those who change take turns to dominate each other.

**Figure 6:**
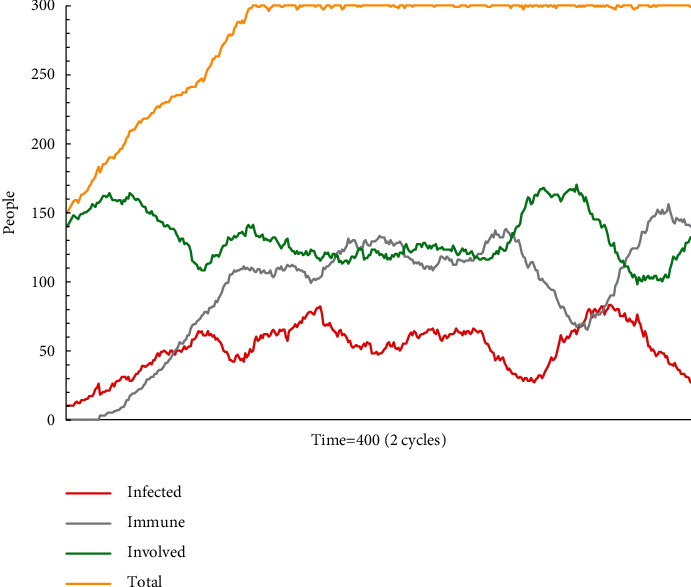
The status monitor of B3.

The results of simulation test B3 and its status monitor are shown in Figure 6. The number of agents in various states varies dramatically. There are several sources of complexity in the information environment: first, the acceptance of escape plan A is 50%, giving it a chance to become the mainstream message; second, employees with rich experience will stick to their own ideas and actions, and transmit their action signals outwardly; third, employees who cannot determine the authenticity of information will hesitate, making wait-and-see strategy as the main behavioural choice.

#### 4.3.3. The Results of the Information with High Acceptance

These models describe the behaviours of the information with high ***acceptance***. In the middle three simulation models, the value of ***change*** swift from 0% to 50%, to 80%; the value of ***acceptance*** is 80%. In these models, the value of ***acceptance*** is 80%. After escape plan A has been disseminated for some time, the proportion of recipients increases significantly and escape plan A has become the mainstream information. The last three simulation models are set as follows: the value of acceptance changes from 20%, to 50%, to 80%; the change of change is 80%.  Table 5 summarises the parameter setting and characteristics of the simulation models.

**Figure 7:**
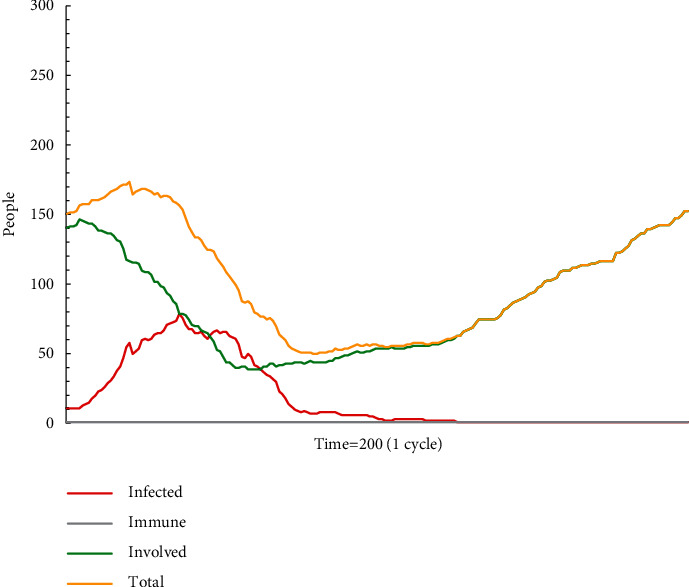
The status monitor of C1.

In simulation test C1 (see Figure 7), the total number of involved populations is increasing, and the number of agents in different states and the rank of proportions fluctuate greatly. The intensity and authority of the information are very high, and the recipients are mostly new employees, who lack experience and knowledge. However, as a large number of the population dies in the emergency (i.e., agents dropping out of the simulation model), the number of potential recipients is limited and the number of recipients of escape plan A decreases accordingly. The withdrawal of recipients and the increase of involved persons may lead to a portion of the new population no longer taking the mainstream escape plan A. This throws the information situation of the whole group into chaos. According to the data, the attenuation of escape plan A occurs in No. 37 time, and the number of recipients of escape plan A is 78.

**Figure 8:**
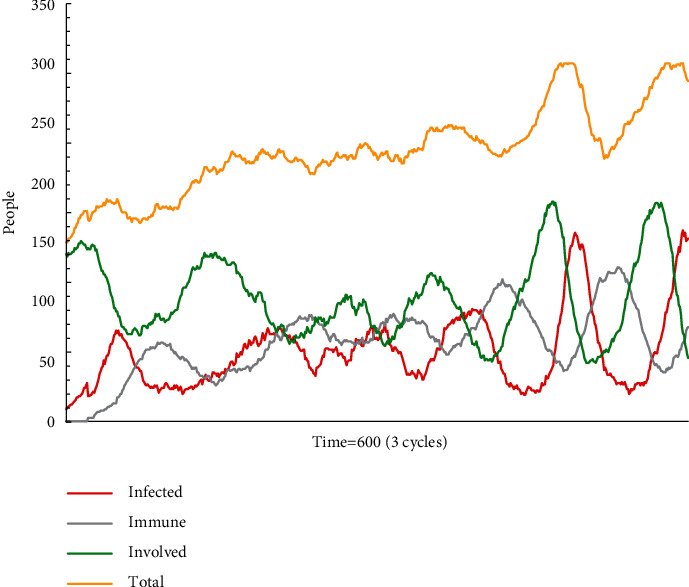
The status monitor of C2.

As shown in Figure 8, the number of agents in all three states regularly fluctuates. The fluctuation in proportion is due to the constant addition of new victims and the quite many grey agents. It results that escape plan A has not been consistently mainstreamed. This simulation model runs for three cycles before showing a clear fluctuation pattern.

**Figure 9:**
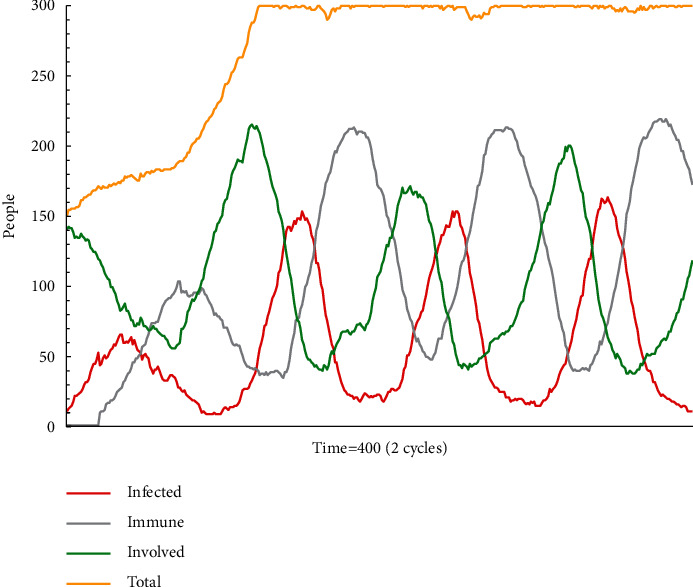
The status monitor of C3.

The result of simulation test C3 is shown in Figure 3. In simulation test C3, the numbers of agents in three states constantly change. During the time window, even though the mainstream information is authoritative, the receiver can insist on their own independent decision, which leads the mainstream information to unable to take the dominant position quickly and comprehensively.  Figure 9 shows the status monitor status of C3.

## 5. Conclusions

### 5.1. Main Findings

***Acceptance, change,*** and ***mainstream***, these three factors are closely related to emergency control. The ***change*** depends on the type of employees and their types of risk attitudes; in other words, during the time window, the ***change*** is an exogenous variable. In sum, considering several factors (including signals, conditional probability, types of risk attitudes, acceptance, change, the proportion of information, and mainstream information), this research uses Bayes conditional probability models and simulations to explore how mainstream information arises, whether mainstream information can be accepted and lead to herd behaviour, as well as the process of herd behaviour. This research also proposes two kinds of control techniques, viz., interfering with information strategy and controlling existing key opinion leaders.

The generation process of mainstream information includes the arising of mainstream information and the acceptance of mainstream information. This research builds a Bayes conditional probability model to describe how individual signals lead to mainstream information and a simulation model to present individual herd behaviour during the generation of mainstream information. The conclusions are as follows.

First, each individual has a fair chance to influence the mainstream information. In emergencies, besides information from media, there is much information on individual decision-making and actions. Once an individual makes decisions, it transmits the relevant signals to others around them.

Second, in emergencies, the order in which individuals make decisions affects the difficulties of decision-making and the likelihood of making rational decisions. Because of the abundance and complexity of information, individuals prefer to delegate decision-making authority to others and accept the behaviour of others as their own norm. A person who takes judgments later in a specific group will pick up on additional signals that will disturb their decision-making. In other words, as compared to persons who make judgments and behaviours rapidly, those who adopt wait-and-see behaviours in the early stage have more difficulty making reasonable decisions.

The third is about the authority of information. If the information lacks authority or has low acceptance, it will soon disappear naturally in the process of dissemination; moderate acceptance will bring confusion to the information status of unexpected events. These two situations can cause a large number of individuals in the group to adopt a wait-and-see strategy and delay making decisions and actions. Only information with high authority can become mainstream and guide individual behaviour.

The fourth is about the importance of individual characteristics, i.e., risk appetite and personal experience. Different types of risk appetite lead to various decisions, which could alleviate the blindness of herd behaviour to some extent, but hesitant individual decision-making processes can affect the implementation of plans. Also, individuals with extensive knowledge and experience are more inclined to stick to their decisions and can help the group to filter out the right or exclude the wrong information, which helps the group behaviour to converge quickly.

### 5.2. Practical Implications

The results and conclusion of the simulation tests suggest that there are two kinds of strategies to control and weaken emergencies, namely, interfering with information strategy and controlling existing key opinion leaders.

The first is the strategy of interfering with information. This strategy is primarily designed to prevent victims from delaying action and hesitating, resulting in difficulties to disseminate and implement rescue plans. ***Acceptance*** regards the intensity and credibility of mainstream information. When ***acceptance*** is low, the information environment is confusing, and no mainstream information exists. If the majority of the victims are new employees or the public who have less professional knowledge, experience, and rescue skills (in models, the setting of the ***change*** is 0%), it means that once adding the mainstream information and action plan in a group, convergent behaviour can occur quickly. This convergent behaviour consists mainly of following the decisions of experienced employees or adopting a wait-and-see strategy. When strengthening the intensity and credibility of the mainstreaming information (e.g., direct orders from superiors, being proposed by authority figures or experts, having scientific backing), the starting time of attenuation would be delayed.

The second is the strategy of controlling existing key opinion leaders. The more experienced the employees are, the more likely they are to stick to their own ideas rather than accepting mainstream information. This can lead to the spread of many individual views within the group, and these views can also influence the action decisions of those around them who are still hesitant. This leads to uncontrolled sources of information within the group, which affects the dissemination of authoritative information. Therefore, to ensure the dissemination and implementation of mainstream information, experienced employees (i.e., key opinion leaders) need to be controlled by persuading them of mainstream messages to them and receiving their acceptance. The opinions of key opinion leaders, whether they support or oppose, have an impact on the action decisions of people around them. Their support for mainstream information allows mainstream information to spread quickly, and their opposition to other information allows other nonauthoritative information to be screened out quickly.

In this study, these two strategies are provided to be useful in detecting and preventing approaches to alleviate individual herd behaviour. In future machine learning models for individual behaviour simulation and prediction, it will be necessary to monitor and collect relevant data in relation to these two strategies, such as the authority of information, the source of information, and the composition of the involved groups.

Several future research directions should be further considered. First, in this research, our context is general emergencies. Future research should differentiate the types of emergencies, for example, by severity (low, medium, high) and by type of emergencies (fire, gas leak, epidemic, earthquake, etc.). Or research should focus on one specific type of emergency. Different types of emergencies can distort or change individual risk perceptions and thus influence decision-making and behaviour. Second, in this simulation model, only individual behavioural information was included in this study. In fact, individuals will also receive a large amount of media information, which will interfere with individual decision-making along with others' behavioural information. The effects of different sources of information should be included in future studies and examine their interactions.

## Figures and Tables

**Table 1:** The implication of types of risk appetite.

Risk appetite (*θ*)	Implication
Risk neutral	Individuals are rational, and the expected value of A and B is equal. From the perspective of probability, there is no deviation or bias in individual decision-making, without over-reaction or under-reaction.

Risk appetite	Individuals tend to collect more information and do comparisons, instead of reacting immediately. Before emergencies, they are in a state of excessive caution and distrust and hold their own decisions. Over time, the reaction gradually weakens.

Risk aversion	Individuals are more sensitive to mainstream information. With the increasing complexities of information, individual responses become more and more intense.

Risk aversion ⟶risk appetite	In the process of interaction, individuals establish the psychological connection of sharing a common destiny, gradually have a trust relationship, and change the type of risk appetite.

Risk appetite ⟶risk aversion	As the situation pressure decreases and individuals can make their own decisions.

**Table 2:** The parameter setting of simulation tests.

No.	Parameter setting (*p*)	Value (%)	Parameter setting (*θ*)	Value (%)
A1	Acceptance	20	Change	0
A1			Change	50
A3			Change	80

B1	Acceptance	50	Change	0
B2			Change	50
B3			Change	80

C1	Acceptance	80	Change	0
C2			Change	50
C3			Change	80

Notes: Number of agents: 150; the proportion of escape plan A: 1/15 (*λ*_*t*=0_  =  1/15).

**Table 3:** Simulation results of A1, A2, and A3.

Setting	A1	A2	A3
Number of agents	150	150	150
Cycle	1 (200 times)	1 (200 times)	1 (200 times)
Infectiousness	20%	20%	20%
Chance of recovery	0%	50%	80%
The intensity of information	Low	Low	Low
The credibility of information	Low authority	Low authority	Low authority
Type of receiver	Lack of experience and knowledge, new employees	Have some experience and knowledge, general staff	Have rich experience and knowledge, able to stick to their own choice, skilled employees
Type of risk appetite	Risk averters	Risk neuter	Risk seeker

**Table 4:** Simulation results of B1, B2, and B3.

Setting	B1	B2	B3
Number of agents	150	150	150
Cycle	1 (200 times)	2 (400 times)	2 (400 times)
Acceptance	50%	50%	50%
The intensity of information	Medium	Medium	Medium
The credibility of information	Medium authority	Medium authority	Medium authority
Change	0%	50%	80%
Type of receiver	Lack of experience and knowledge, new employees	Have some experience and knowledge, general staff	Have rich experience and knowledge, able to stick to their own choice, skilled employees
Type of risk appetite	Risk averters	Risk neuter	Risk seeker

**Table 5:** Simulation results of C1, C2, and C3.

Setting	C1	C2	C3
Number of agents	150	150	150
Cycle	1 (200 times)	3 (600 times)	2 (400 times)
Acceptance	50%	50%	50%
The intensity of mainstream information	Strong	Strong	Strong
The credibility of mainstream information	High authority	High authority	High authority
Change	0%	50%	80%
Type of receiver	Lack of experience and knowledge, new employees	Have some experience and knowledge, general staff	Have rich experience and knowledge, able to stick to their own choice, skilled employees
Type of risk appetite	Risk averters	Risk neuter	Risk seeker

## Data Availability

The data used to support the findings of this study are included within the article.
